# Supplementary material for: Disease trajectory browser for exploring temporal, population-wide disease progression patterns in 7.2 million Danish patients
Source: Nat Commun. 2020 Oct 2;11:4952. doi: 10.1038/s41467-020-18682-4 (PMC7532164; doi:10.1038/s41467-020-18682-4)
Supplement: Supplementary file 2 — Description of Additional Supplementary Files [file 41467_2020_18682_MOESM2_ESM.docx]

Supplementary Information to:

**Disease trajectory browser for exploring temporal, population-wide disease progression patterns in 7.2 million Danish patients

Siggaard et al.**

File Name: Supplementary Data 1

Description:

The Supplementary Data 1 contains the disease pairs and the statistical summary for the entire Danish population. The diseases (Columns D1 and D2) are encoded in International Statistical Classification of Diseases and Related Health Problems version 10 (ICD-10).
**D1:** The first disease occurring in a disease pair.
**D2:** The second disease occurring in a disease pair.
**RR:** The relative risk. It includes all values independently of their relative risk. For visualisation purposes the disease pairs that have RR < 1 are excluded from the browser.
**direction_yes_no**: Encodes the directionality of the pair. If the value == 1 the pair has statistical directionality, meaning that D1 occurs statistically significant more often before D2. A value == -1 means that the pair has no directionality implying that either of the diseases occur significantly more often before the other disease.
**p5years**: Refers to the uncorrected p-value that the pair has in comparison to the background population.
**p.value.direction**: Refers to the p-value from the binomial test to compute the directionality of the disease pair.
**AGE_AT_DISEASE**: Represents the average age of the population in days that follows the disease pair at the time of the diagnosis of D1.
**counts:** This is the total counts of people that have been diagnosed with both diseases (D1 and D2).
**female_counts:** Represent the counts of how many women follow the disease pair. This is a subset of the “counts” column.
**CODE_DIFF_DAYS**: Represents the average time span in days between the first diagnosis D1 and D2 for all individuals that follow the disease pair. If there is no directionality the value is set to nan.
**death_counts**: This counts the individuals who died within 5 years of the second diagnosis (D2). Individuals who died later are not included in the counts.

If there are less than 20 people, the value is set to nan to protect data privacy.
